# Effects of fecal microbiota transplantation on glycemic and lipid profiles in overweight or obese patients with metabolic disorders: a systematic review and meta-analysis

**DOI:** 10.3389/fendo.2025.1737543

**Published:** 2025-12-15

**Authors:** Yuxin Zhang, Jingsi Cao, Yanyan Wang, Xuechun Fan, Ruixue Deng, Jia Mi

**Affiliations:** 1 College of Traditional Chinese Medicine, Changchun University of Chinese Medicine, Changchun, Jilin, China; 2 The Affiliated Hospital to Changchun University of Chinese Medicine, Changchun University of Chinese Medicine, Changchun, Jilin, China

**Keywords:** fecal microbiota transplantation, metabolic syndrome, obesity, insulin resistance, meta-analysis

## Abstract

Obesity and its associated metabolic disorders (such as type 2 diabetes, metabolic syndrome, and NAFLD/MASLD) represent a global health challenge. Fecal microbiota transplantation (FMT), as a therapy regulating the gut microbiome, has demonstrated inconsistent clinical efficacy. This systematic review aims to evaluate the impact of FMT on key indicators of glucose and lipid metabolism in overweight/obese adults with metabolic diseases. We systematically searched PubMed, Embase, Cochrane, and Web of Science databases up to September 28, 2025, to identify randomized controlled trials evaluating FMT for obesity and metabolic disorders. Data were pooled using a random-effects model, with primary outcomes being changes in BMI, HOMA-IR, and HbA1c relative to baseline. A total of 11 RCTs (320 participants) were included. The primary analysis showed that FMT intervention demonstrated a trend toward improvement in the primary outcome measures, BMI (MD: -0.65, *p* = 0.070) and HOMA-IR (MD: -0.64, *p* = 0.062), but these trends did not reach statistical significance. There was no significant effect on HbA1c (MD: 0.06, *p* = 0.742). However, this negative conclusion based on the conventional assumption (Corr = 0.5) exhibited high instability: sensitivity analysis revealed that FMT’s improvement effects on BMI became statistically significant (*p* = 0.010) when the correlation coefficient (Corr) used to estimate the standard deviation of change was adjusted to 0.75. Furthermore, meta-regression analysis revealed that treatment regimen, follow-up duration, and patient baseline characteristics were significantly associated with HbA1c efficacy. Based on the current “low” certainty evidence, the overall improvement of metabolic parameters in overweight or obese patients with metabolic diseases following FMT did not reach statistical significance in the primary analysis. However, the robustness of this negative finding is limited, exhibiting high sensitivity to statistical hypotheses and likely being significantly confounded by methodological factors (e.g., administration protocols) and donor variability. Consequently, the true efficacy of FMT for treating systemic metabolic disorders remains uncertain. Future trials should shift toward precision medicine, prioritizing the standardization of donor selection and optimization of administration protocols.

**Systematic Review Registration:**

https://www.crd.york.ac.uk/prospero/display_record.php?ID=CRD420251172011, identifier CRD420251172011.

## 1 Introduction

Obesity and its associated metabolic disorders, including metabolic syndrome, type 2 diabetes (T2DM), and metabolic dysfunction-associated steatohepatitis (MASLD)/non-alcoholic fatty liver disease (NAFLD), have emerged as one of the most severe global health epidemics of the 21st century. The impact of these diseases is vast: the prevalence of metabolic syndrome now rivals that of obesity and diabetes, affecting up to 45% of the global population (1); T2DM affects hundreds of millions worldwide, with cases projected to double by 2030 (2); and NAFLD/MASLD has become the most common chronic liver disease globally, with prevalence soaring to 71–92% among obese individuals (3). These conditions not only substantially increase morbidity and mortality risks but may also progress to cirrhosis, hepatocellular carcinoma, or cardiovascular disease, driving sustained healthcare expenditures (4). However, conventional lifestyle interventions and pharmacological treatments face limitations in efficacy, cost-effectiveness, or long-term adherence. Consequently, exploring innovative therapeutic approaches has become an urgent public health imperative (5).

In recent years, the gut microbiota has been recognized as a key regulator of host metabolism and energy homeostasis. Increasing evidence indicates that dysbiosis represents one of the core pathophysiological mechanisms underlying obesity, insulin resistance, and related metabolic disorders (6–8). Consequently, modulating the gut microbiota to restore its healthy balance is viewed as a highly promising new therapeutic target (9). As a potent intervention capable of reconstructing the gut microbiome, FMT—involving the transplantation of gut microbiota from healthy donors—has demonstrated remarkable success in treating conditions such as recurrent Clostridioides difficile infection (10, 11). This success has sparked significant interest in its potential application for treating metabolic diseases.

However, as FMT transitions from animal models to clinical applications, its actual efficacy in treating metabolic diseases remains highly controversial. Pioneering trials in this field initially reported that FMT significantly improved insulin sensitivity in patients with metabolic syndrome, driving the advancement of FMT as a treatment for metabolic disorders (12, 13). Yet these positive signals have not been consistently replicated in subsequent studies. Conversely, numerous subsequent randomized controlled trials (RCTs) evaluating key metabolic markers have yielded a multitude of conflicting conclusions, reporting either no effect from FMT or even slight deterioration (14–16). This widespread heterogeneity in outcomes likely stems from substantial variations in donor selection, intervention protocols, administration routes, and baseline characteristics of participants. These differences make it challenging for clinicians and researchers to reliably assess the true efficacy of FMT. Consequently, the contradictory nature of the existing evidence significantly limits the objective evaluation of its clinical value.

Therefore, given the current inconsistencies in clinical evidence and the limitations of individual studies, a comprehensive systematic review and meta-analysis are warranted. This study aims to objectively evaluate the true impact of FMT on key metabolic indicators in obese and metabolic disease populations by integrating data from existing relevant RCTs to provide a more precise and robust effect size estimate. The findings will provide critical evidence-based medical support for the clinical application of FMT in this field and future research directions.

## 2 Method

This systematic review and meta-analysis were conducted in accordance with the PRISMA statement (17, 18). The study protocol has been registered with PROSPERO (registration number: CRD420251172011).

### 2.1 Search strategy and study selection

We conducted a systematic search of PubMed, Embase, Cochrane, and Web of Science databases from their inception to September 28, 2025. The complete search strategy is detailed in the **Supplementary Appendix (Supplementary Table S1)**. The search was limited to English-language publications. Additionally, we identified other relevant studies by reviewing the reference lists of included studies. Two researchers independently performed a two-stage screening process: first, screening titles and abstracts, and then evaluating the full texts of potentially eligible studies. All disagreements were resolved through discussion or third-party adjudication.

### 2.2 Inclusion and exclusion criteria

Studies must meet the following criteria for inclusion: (1) Study design: Randomized controlled trials (RCTs); (2) Subjects: Adults aged 18 years or older who are overweight or obese (BMI ≥ 25 kg/m²) and have one or more of the following metabolic conditions: type 2 diabetes, metabolic syndrome, insulin resistance, non-alcoholic fatty liver disease (NAFLD)/metabolic dysfunction-associated steatohepatitis (MASLD), or impaired glucose tolerance; (3) Intervention: Allogeneic fecal microbiota transplantation (FMT) from healthy lean donors; (4) Control measures: Autologous FMT (using the patient’s own stool), placebo (e.g., saline infusion or inactive capsules), and conventional treatment (e.g., metformin alone). Non-randomized studies, observational studies, animal experiments, and reviews were excluded. (5) Outcome measures: Studies reported at least one primary outcome measure of interest to this research.

### 2.3 Data extraction and risk of bias assessment

Two researchers independently extracted data using standardized forms and cross-checked each other’s work. Extracted information included study characteristics, baseline participant data, intervention details, and outcome data. Subsequently, these same two researchers independently assessed the methodological quality of each included RCT using the Cochrane Risk of Bias tool, version 2 (RoB 2). The assessment covered five domains of bias risk: randomization process, deviation from the intervention, missing outcome data, outcome measurement, and selective reporting of results. Based on the assessment, the overall risk of bias for each study was classified as “low risk, “ “some concern, “ or “high risk.” Throughout the data extraction and assessment process, any disagreements were resolved through discussion or by involving a third-party researcher.

### 2.4 Outcome measures

The primary outcome measures of this study include changes in BMI, HOMA-IR, and HbA1c relative to baseline. Secondary outcome measures include alterations in lipid profiles (total cholesterol (TC), low-density lipoprotein (LDL), high-density lipoprotein (HDL), and triglycerides (TG)).

### 2.5 Statistical analysis

All quantitative analyses were performed using the meta package in R software (version 4.5.1). This analysis utilized baseline and trial endpoint data extracted from individual studies. The standard deviation of change (SDchange) was estimated using the formula recommended in the Cochrane Manual, with the correlation coefficient (Corr) between baseline and endpoint set at 0.5 for the primary analysis (19). All outcome measures were standardized to a consistent unit of measurement. Data were pooled using a random-effects model and restricted maximum likelihood (REML) estimation. Depending on the outcome unit, either the mean difference (MD) or standardized mean difference (SMD) was selected as the pooled effect measure, with its 95% confidence interval (CI) calculated. Heterogeneity between studies was assessed using Cochran’s Q test and quantified with the I² statistic (I² > 50% indicating substantial heterogeneity). Results were visualized using forest plots. Sources of heterogeneity were explored through pre-specified subgroup analyses based on the following factors: control group type (compared to autologous FMT vs. compared to placebo/other), administration regimen (single vs. multiple doses), follow-up duration (≤12 weeks vs. >12 weeks), and patient population (non-diabetic vs. diabetic). Studies lacking specific subgroup classification information were excluded from this subgroup analysis.

To assess the robustness of the results, two sensitivity analyses were performed: 1) Leave-One-Out analysis; 2) Re-running the primary analysis after modifying the correlation coefficient (Corr) used to estimate SDchange to 0.25 and 0.75, respectively. For outcomes with at least 10 included studies, publication bias was assessed using funnel plots and Egger’s test. Finally, the certainty of evidence for primary outcomes was graded using the GRADE approach.

## 3 Results

### 3.1 Search strategy

**Figure 1:**
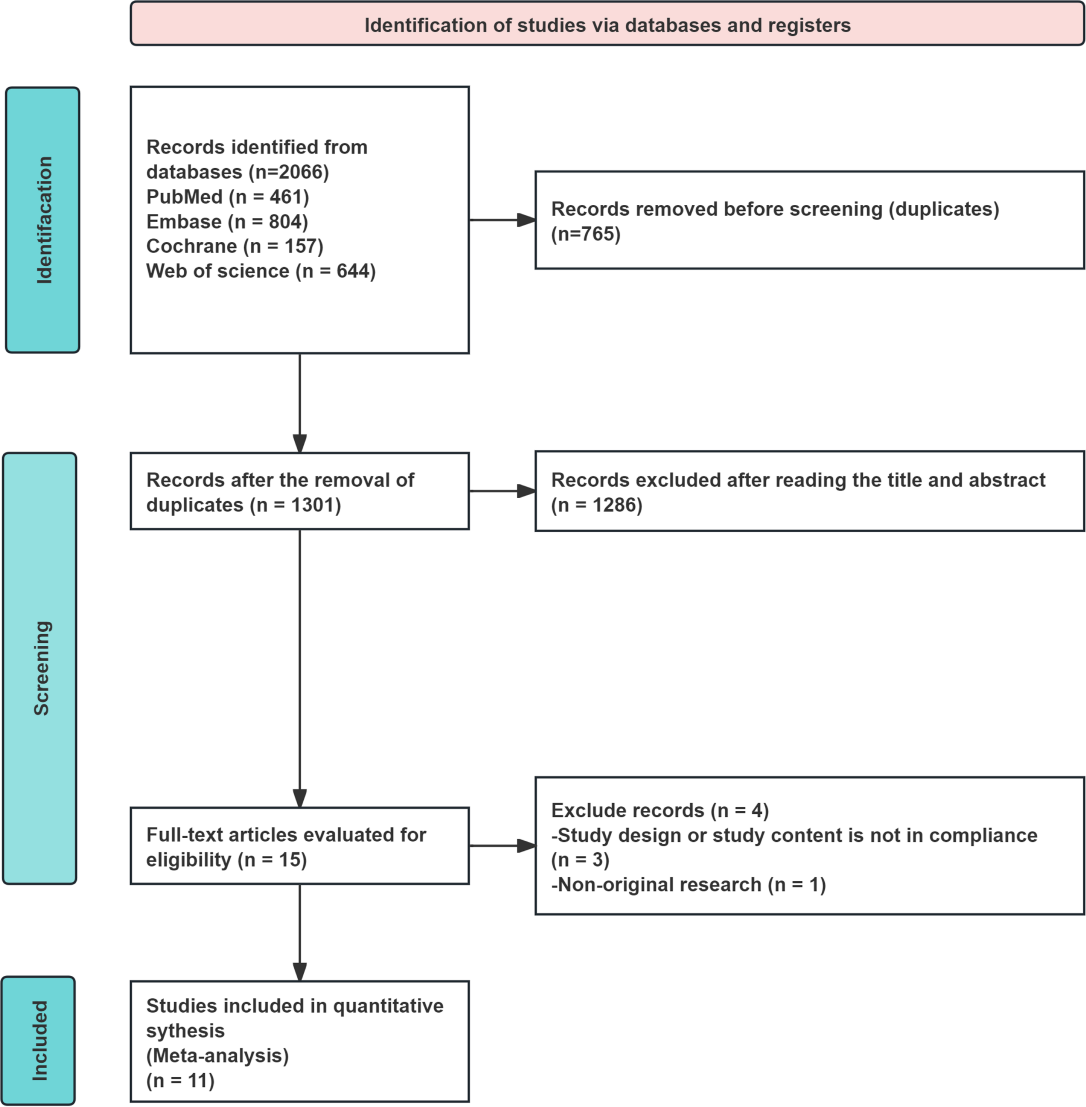
PRISMA flow diagram of the study selection process.

The initial database search identified a total of 2, 066 publications: 461 from PubMed, 644 from Web of Science, 804 from Embase, and 157 from the Cochrane Library. After removing 765 duplicate records, we screened the titles and abstracts of the remaining 1, 301 publications. Of these, 1, 286 were excluded due to clear irrelevance to the topic. We conducted full-text assessments of 15 potentially eligible studies. Upon review, four were excluded due to non-compliant study designs. Ultimately, 11 RCTs meeting all criteria were included in this systematic review and meta-analysis. The detailed screening process is illustrated in **Figure 1** (PRISMA flow diagram).

### 3.2 Trial characteristics and subject demographics

This meta-analysis included 11 randomized controlled trials (RCTs) (12, 15, 16, 20–27), with detailed characteristics summarized in **Table 1**. These studies initially randomized 340 participants, with data from 320 participants ultimately included in the analysis: 177 received FMT intervention and 143 were in the corresponding control groups. Geographically, three studies (27.3%) were conducted in the Netherlands (12, 20, 26), three (27.3%) in Canada (15, 24, 27), and the remaining five (45.5%) in China (23), Hong Kong SAR, China (25), Spain (21), Brazil (21), and the United States (16).

**Table 1:** Characteristics of included randomized controlled trials.

Study information		Sample size		Participant baseline characteristics		Intervention & comparator		Follow-up timepoints
Study	Country	Randomized (N)	Analyzed (N)	Population	Key baseline values (FMT/control)	FMT group details	Control group details	
Groenewegen, 2025 (20)	Netherlands	FMT: 10 Control: 10	FMT: 10 Control: 10	Dx: MASLD Sex (M/F): 4/6 (FMT), 4/6 (Control) Age (yrs): 62.3 ± 4.9 (FMT), 63.2 ± 5.1 (Control)	BMI: 34.0 ± 6.8/34.0 ± 6.4 HOMA-IR: 6.2 ± 2.6/10.4 ± 7.3 HbA1c: NR	Route: Duodenal endoscopy Donor: Single (2 donors total) Dose: Frozen, 60g feces/198ml Regimen: 3 doses (Wks 0, 3, 6)	Type: Autologous FMT Matching: Matched route, volume, schedule	12 weeks
Gómez-Pérez, 2024 (21)	Spain	FMT: 7 Control: 7	FMT: 6 Control: 6	Dx: T2D (on metformin) Sex (M/F): NR Age (yrs): NR	BMI: NR HOMA-IR: NR HbA1c: NR	Route: Oral capsules Donor: Pooled (2 donors) Dose: Lyophilized (NR weight) Regimen: Single dose	Type: Placebo Matching: Matched route, dose, schedule	4, 12 weeks
da Ponte Neto, 2023 (22)	Brazil	FMT: 16 Control: 16	FMT: 15 Control: 13	Dx: MetS & Class II Obesity Sex (M/F): 0/16 (FMT), 0/16 (Control) Age (yrs): 55.2 ± 10.2 (FMT), 53.6 ± 13.1 (Control)	BMI: 36.7 ± 2.9/35.7 ± 2.2 HOMA-IR: NR HbA1c: 6.8 ± 1.1/7.0 ± 2.0	Route: Upper GI endoscopy Donor: Single Dose: Fresh, 200g feces/500ml Regimen: Single infusion	Type: Placebo (saline) Matching: Matched route, volume, schedule	6 wks, 6 mos, 1 yr
Wu, 2023 (23)	China	FMT: 19 Control: 12	FMT: 17 Control: 12	Dx: T2D Sex (M/F): NR Age (yrs): NR	BMI: 27.2 ± 1.0/27.5 ± 0.9 HOMA-IR: 6.7 ± 2.9/4.0 ± 1.4 HbA1c: 10.8 ± 1.9/8.6 ± 1.1	Route: Nasojejunal tube Donor: Pooled (10 donors) Dose: 50g sludge/200ml Regimen: Single infusion	Type: Active (Metformin) Matching: Not matched	4 weeks
Ghorbani, 2022 (24)	Canada	FMT: 15 Control: 13	FMT: 15 Control: 13	Dx: Severe obesity & IR Sex (M/F): 3/12 (FMT), 3/10 (Control) Age (yrs): 45.7 ± 12.1 (FMT), 47.2 ± 10.6 (Control)	BMI: 44.1 ± 6.8/45.4 ± 7.5 HOMA-IR: 4.3 ± 1.1/6.9 ± 5.7 HbA1c: 5.0 ± 0.0/5.0 ± 0.1	Route: Colonoscopy Donor: Single (3 donors total) Dose: Frozen, 150g feces/300ml Regimen: Single dose	Type: Autologous FMT Matching: Matched route, volume, schedule	1, 3 months
Ng, 2022 (25)	Hong Kong SAR, China	FMT: 41 Control: 20	FMT: 41 Control: 20	Dx: Obesity & T2D Sex (M/F): 29/12 (FMT), 14/6 (Control) Age (yrs): NR	BMI: NR HOMA-IR: NR HbA1c: NR	Route: EGD infusion Donor: Pooled Dose: Frozen, 50g feces/100-200ml Regimen: 4 doses (q4wks)	Type: Placebo (saline) Matching: Matched route, volume, schedule	24 weeks
Witjes, 2020 (26)	Netherlands	FMT: 10 Control: 11	FMT: 10 Control: 11	Dx: NAFLD & NASH Sex (M/F): NR Age (yrs): 51.2 ± 6.6 (FMT), 48.5 ± 10.2 (Control)	BMI: 31.7 ± 3.5/31.5 ± 4.8 HOMA-IR: NR HbA1c: NR	Route: Duodenal infusion Donor: Single Dose: Fresh, NR Regimen: 3 doses (Wks 0, 8, 16)	Type: Autologous FMT Matching: Matched route, volume, schedule	24 weeks
Craven, 2020 (15)	Canada	FMT: 15 Control: 6	FMT: 15 Control: 6	Dx: NAFLD (AASLD) Sex (M/F): 5/10 (FMT), 1/5 (Control) Age (yrs): 47.6 ± 14.9 (FMT), 57.5 ± 13.0 (Control)	BMI: 36.3 ± 5.0/37.4 ± 9.5 HOMA-IR: 3.5 ± 1.3/4.4 ± 2.1 HbA1c: 6.3 ± 0.9/6.4 ± 1.0	Route: Duodenal endoscopy Donor: Single (3 donors total) Dose: Fresh, 2g feces/125ml Regimen: Single infusion	Type: Autologous FMT Matching: Matched route, dose, schedule	6 weeks, 6 months
Yu, 2020 (16)	USA	FMT: 12 Control: 12	FMT: 11 Control: 12	Dx: Obesity & IR Sex (M/F): 4/8 (FMT), 3/9 (Control) Age (yrs): 42.5 ± 8.4 (FMT), 38.5 ± 8.8 (Control)	BMI: 38.8 ± 6.7/41.3 ± 5.1 HOMA-IR: 3.5 ± 1.4/3.5 ± 1.9 HbA1c: 5.6 ± 0.2/5.5 ± 0.3	Route: Oral capsules Donor: Single Dose: Frozen, ~24g material/dose Regimen: Multi-dose (6 wks)	Type: Placebo Matching: Matched route, dose, schedule	6, 12 weeks
Vrieze, 2012 (12)	Netherlands	FMT: 9 Control: 9	FMT: 8 Control: 8	Dx: MetS Sex (M/F): 9/0 (FMT), 9/0 (Control) Age (yrs): 47 ± 4 (FMT), 53 ± 3 (Control)	BMI: 35.7 ± 1.5/35.6 ± 1.5 HOMA-IR: NR HbA1c: 39 ± 1.1/40 ± 1.5 (mmol/mol)	Route: Duodenal tube Donor: NR (lean donors) Dose: Fresh, in 500ml saline Regimen: Single infusion	Type: Autologous FMT Matching: Matched route, preparation, schedule	6 weeks
Mocanu, 2021 (27)	Canada	FMT: 34 Control: 36	FMT: 29 Control: 32	Dx: Severe obesity & MetS Sex (M/F): 3/26 (FMT), 7/25 (Control) Age (yrs): 47.3 ± 11.0 (FMT), 48.4 ± 9.6 (Control)	BMI: 46.2 ± 6.6/44.5 ± 7.2 HOMA-IR: 2.8 ± 1.9/3.2 ± 2.4 HbA1c: 5.8 ± 0.6/5.8 ± 0.7	Route: Oral capsules Donor: Pooled (4 donors) Dose: 20 caps from 50g feces Regimen: Single dose	Type: Placebo Matching: Matched route, dose, schedule	2, 6, 12 weeks

All studies utilized screened, healthy, lean donors. Values for participant characteristics and baseline data are presented as Mean ± SD unless otherwise specified. AASLD, American Association for the Study of Liver Diseases; ADA, American Diabetes Association; BMI, Body Mass Index; Dx, Diagnosis; EGD, Esophagogastroduodenoscopy; FMT, Fecal Microbiota Transplantation; GI, Gastrointestinal; HOMA-IR, Homeostatic Model Assessment of Insulin Resistance; IR, Insulin Resistance; MASLD, Metabolic Dysfunction-Associated Steatotic Liver Disease; MetS, Metabolic Syndrome; NAFLD, Non-alcoholic Fatty Liver Disease; NASH, Non-alcoholic Steatohepatitis; NR, Not Reported; q4wks, every 4 weeks; T2D, Type 2 Diabetes.

All studies recruited adults with overweight or obesity accompanied by metabolic diseases. Among the 11 studies included in the pooled analysis, the median age of participants was 50.0 years (IQR: 47.5–53.0 years; range: 38.5–63.2 years), and the median baseline body mass index (BMI) was 36.2 kg/m² (IQR: 34.0–40.1 kg/m²; range: 27.2–46.2 kg/m²).

Regarding intervention methods, seven studies (63.6%) employed a single-dose FMT regimen, while four studies (36.4%) utilized multiple doses. The primary route of administration was via the upper gastrointestinal tract (10 studies, 90.9%), including oral capsules, nasojejunal tubes, and various endoscopic duodenal/jejunal infusions; Only one study (9.1%) employed a colonoscopic route. Control group designs varied: five studies (45.5%) used autologous FMT as a control, five (45.5%) used a placebo (e.g., inert capsules or saline), and one (9.1%) used an active drug (metformin) as a control. The median follow-up duration for primary outcome measures across studies was 12 weeks (IQR: 12–24 weeks; range: 2–52 weeks).

### 3.3 Bias risk assessment

**Figure 2:**
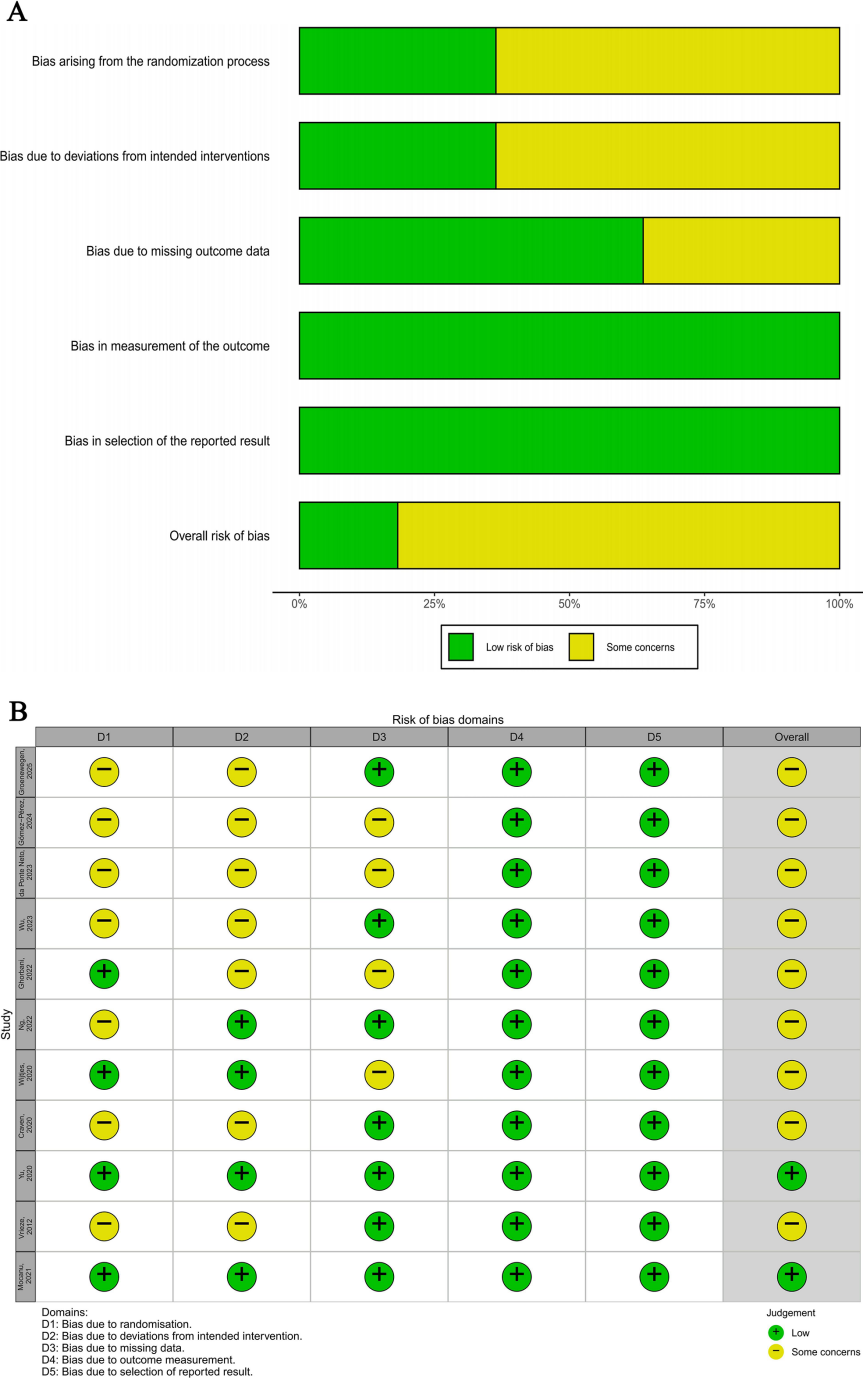
Risk of bias (RoB 2) summary for included RCTs. **(A)** Summary of risk of bias for each domain, presented as percentages. **(B)** Risk of bias traffic light plot for each included study.

Risk of Bias Assessment Methodological quality assessments were conducted for the 11 included RCTs (**Figure 2**). Regarding overall risk of bias, 18.2% (2/11) of studies were rated as low risk, while the remaining 81.8% (9/11) were rated as “some concerns.” None of the studies was rated as high risk.

Regarding specific domains of bias: For randomization processes, 36.4% (4/11) of studies were at low risk, while 63.6% (7/11) raised some concerns. Concerning deviation from the intervention, 36.4% (4/11) were at low risk, and 63.6% (7/11) raised some concerns. Regarding missing outcome data, 63.6% (7/11) of studies were rated as low risk. Notably, all included studies (100%) demonstrated low risk of bias in both outcome measurement and selection of reported results.

### 3.4 Effects on BMI

**Figure 3:**
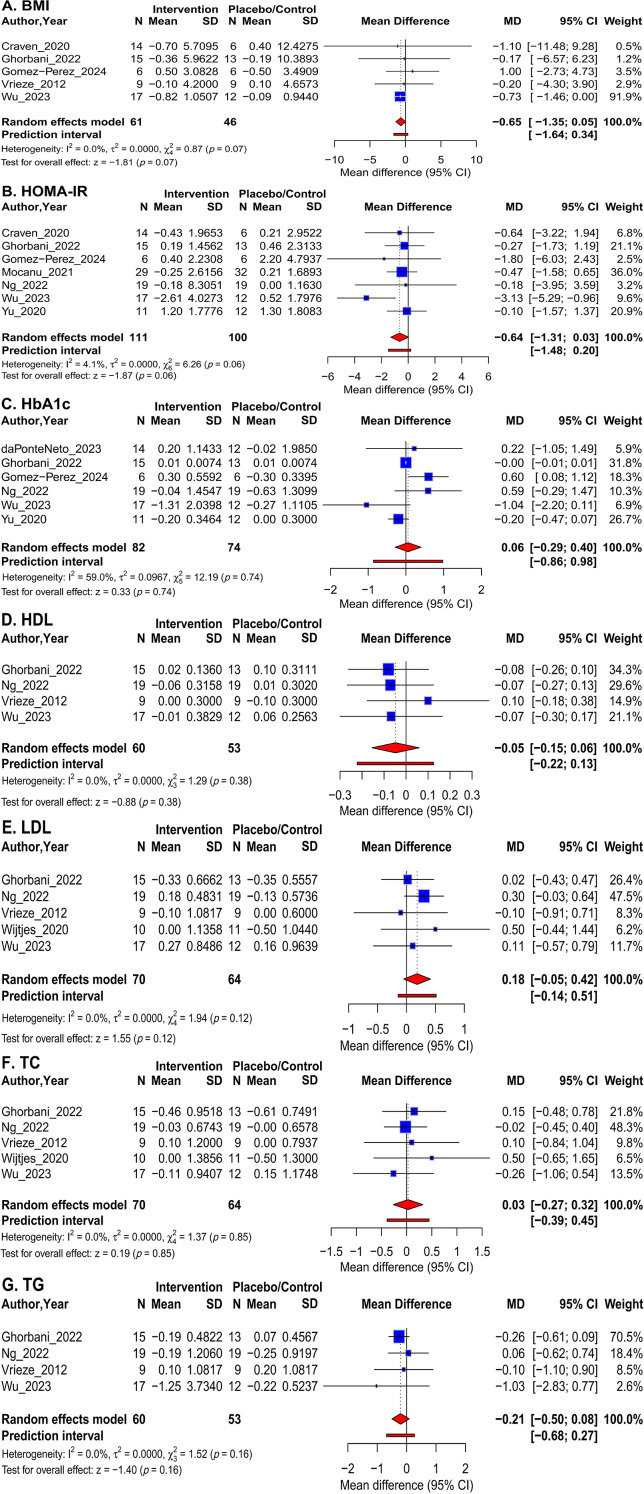
Forest plots of the meta-analysis on the effect of FMT versus control on primary and secondary outcomes: **(A)** BMI; **(B)** HOMA-IR; **(C)** HbA1c; **(D)** HDL; **(E)** LDL; **(F)** TC; and **(G)** TG.

Pooled analysis of data from five trials showed that the overall effect of FMT intervention on BMI did not reach statistical significance compared with the control group (MD: -0.65 [95% CI: -1.35; 0.05]; *p* = 0.070, I² = 0.0%, n=107) (**Figure 3A**). Subgroup analyses (**Table 2**) revealed no significant differences between subgroups based on control group type (*p* = 0.822), administration regimen (*p* = 0.822), follow-up duration (*p* = 0.399), or patient population (*p* = 0.822). Furthermore, meta-regression analysis revealed no significant linear relationship between BMI change and treatment regimen (*p* = 0.851), duration of follow-up (*p* = 0.462), or patient population (*p* = 0.851) (**Table 3**).

**Table 2:** Subgroup analysis for primary and secondary outcomes (based on the primary analysis assumption, Corr=0.5).

Outcome	Subgroup	Level	Trials	Participants	MD (95% CI)	*p*-value	lI²
BMI	Overall		5	107	-0.65 (-1.35, 0.05)	0.070	0.0%
	Control_Type	vs. Autologous FMT	3	66	-0.28 (-3.56, 2.99)	0.866	0.0%
		vs. Placebo/Other	2	41	-0.67 (-1.38, 0.05)	0.069	0.0%
		*p for subgroup difference*			*0.822*		
	Dosage	Single	3	66	-0.28 (-3.56, 2.99)	0.866	0.0%
		Multiple	2	41	-0.67 (-1.38, 0.05)	0.069	0.0%
		*p for subgroup difference*			*0.822*		
	Followup	≤12 weeks	3	67	-0.72 (-1.43, 0.00)	0.051	0.0%
		>12 weeks	2	40	0.70 (-2.52, 3.92)	0.668	0.0%
		*p for subgroup difference*			*0.399*		
	Population_Grouped	Non-Diabetic Population	3	66	-0.28 (-3.56, 2.99)	0.866	0.0%
		Diabetic Population	2	41	-0.67 (-1.38, 0.05)	0.069	0.0%
		*p for subgroup difference*			*0.822*		
HOMA_IR	Overall		7	211	-0.64 (-1.31, 0.03)	0.062	4.1%
	Control_Type	vs. Autologous FMT	2	48	-0.36 (-1.63, 0.91)	0.578	0.0%
		vs. Placebo/Other	5	163	-0.94 (-2.14, 0.26)	0.124	32.7%
		*p for subgroup difference*			*0.514*		
	Dosage	Single	4	109	-0.24 (-1.17, 0.69)	0.607	0.0%
		Multiple	3	102	-1.59 (-3.53, 0.35)	0.109	57.3%
		*p for subgroup difference*			*0.221*		
	Followup	≤12 weeks	3	72	-1.21 (-3.09, 0.67)	0.208	61.6%
		>12 weeks	4	139	-0.44 (-1.29, 0.41)	0.309	0.0%
		*p for subgroup difference*			*0.465*		
	Population_Grouped	Non-Diabetic Population	4	132	-0.34 (-1.07, 0.39)	0.358	0.0%
		Diabetic Population	3	79	-2.22 (-4.08, -0.37)	0.019	0.0%
		*p for subgroup difference*			*0.064*		
HbA1c	Overall		6	156	0.06 (-0.29, 0.40)	0.742	59.0%
	Control_Type	vs. Placebo/Other	5	128	0.08 (-0.44, 0.61)	0.754	67.0%
		vs. Autologous FMT	1	28	-0.00 (-0.01, 0.01)	1.000	-
		*p for subgroup difference*			*0.754*		
	Dosage	Single	4	115	-0.02 (-0.15, 0.11)	0.741	25.0%
		Multiple	2	41	-0.14 (-1.74, 1.46)	0.866	84.5%
		*p for subgroup difference*			*0.887*		
	Followup	≤12 weeks	3	78	-0.22 (-0.48, 0.03)	0.084	17.8%
		>12 weeks	3	78	0.29 (-0.17, 0.75)	0.218	70.4%
		*p for subgroup difference*			*0.056*		
	Population_Grouped	Diabetic Population	4	105	0.20 (-0.50, 0.90)	0.582	55.8%
		Non-Diabetic Population	2	51	-0.05 (-0.23, 0.12)	0.543	54.0%
		*p for subgroup difference*			*0.496*		
HDL	Overall		4	113	-0.05 (-0.15, 0.06)	0.380	0.0%
	Control_Type	vs. Autologous FMT	2	46	-0.02 (-0.19, 0.14)	0.799	11.5%
		vs. Placebo/Other	2	67	-0.07 (-0.22, 0.08)	0.363	0.0%
		*p for subgroup difference*			*0.673*		
	Dosage	Single	3	84	-0.04 (-0.16, 0.08)	0.488	0.0%
		Multiple	1	29	-0.07 (-0.30, 0.17)	0.569	-
		*p for subgroup difference*			*0.852*		
	Followup	>12 weeks	2	66	-0.08 (-0.21, 0.06)	0.266	0.0%
		≤12 weeks	2	47	0.00 (-0.18, 0.18)	0.985	0.0%
		*p for subgroup difference*			*0.495*		
	Population_Grouped	Non-Diabetic Population	2	46	-0.02 (-0.19, 0.14)	0.799	11.5%
		Diabetic Population	2	67	-0.07 (-0.22, 0.08)	0.363	0.0%
		*p for subgroup difference*			*0.673*		
LDL	Overall		5	134	0.18 (-0.05, 0.42)	0.120	0.0%
	Control_Type	vs. Autologous FMT	3	67	0.07 (-0.30, 0.43)	0.713	0.0%
		vs. Placebo/Other	2	67	0.26 (-0.04, 0.57)	0.086	0.0%
		*p for subgroup difference*			*0.417*		
	Dosage	Single	4	105	0.19 (-0.05, 0.44)	0.123	0.0%
		Multiple	1	29	0.11 (-0.57, 0.79)	0.757	-
		*p for subgroup difference*			*0.812*		
	Followup	>12 weeks	2	66	0.20 (-0.07, 0.47)	0.143	0.0%
		≤12 weeks	3	68	0.13 (-0.32, 0.59)	0.563	0.0%
		*p for subgroup difference*			*0.801*		
	Population_Grouped	Non-Diabetic Population	3	67	0.07 (-0.30, 0.43)	0.713	0.0%
		Diabetic Population	2	67	0.26 (-0.04, 0.57)	0.086	0.0%
		*p for subgroup difference*			*0.417*		
TC	Overall		5	134	0.03 (-0.27, 0.32)	0.848	0.0%
	Control_Type	vs. Autologous FMT	3	67	0.20 (-0.28, 0.67)	0.418	0.0%
		vs. Placebo/Other	2	67	-0.08 (-0.45, 0.30)	0.694	0.0%
		*p for subgroup difference*			*0.379*		
	Dosage	Single	4	105	0.07 (-0.24, 0.39)	0.647	0.0%
		Multiple	1	29	-0.26 (-1.06, 0.54)	0.523	-
		*p for subgroup difference*			*0.446*		
	Followup	>12 weeks	2	66	0.03 (-0.32, 0.38)	0.864	0.0%
		≤12 weeks	3	68	0.02 (-0.51, 0.56)	0.930	0.0%
		*p for subgroup difference*			*0.984*		
	Population_Grouped	Non-Diabetic Population	3	67	0.20 (-0.28, 0.67)	0.418	0.0%
		Diabetic Population	2	67	-0.08 (-0.45, 0.30)	0.694	0.0%
		*p for subgroup difference*			*0.379*		
TG	Overall		4	113	-0.21 (-0.50, 0.08)	0.163	0.0%
	Control_Type	vs. Autologous FMT	2	46	-0.24 (-0.57, 0.09)	0.148	0.0%
		vs. Placebo/Other	2	67	-0.15 (-1.00, 0.69)	0.723	18.4%
		*p for subgroup difference*			*0.845*		
	Dosage	Single	3	84	-0.19 (-0.48, 0.11)	0.219	0.0%
		Multiple	1	29	-1.03 (-2.83, 0.77)	0.263	-
		*p for subgroup difference*			*0.365*		
	Followup	>12 weeks	2	66	-0.19 (-0.50, 0.12)	0.220	0.0%
		≤12 weeks	2	47	-0.32 (-1.19, 0.55)	0.475	0.0%
		*p for subgroup difference*			*0.792*		
	Population_Grouped	Non-Diabetic Population	2	46	-0.24 (-0.57, 0.09)	0.148	0.0%
		Diabetic Population	2	67	-0.15 (-1.00, 0.69)	0.723	18.4%
		*p for subgroup difference*			*0.845*		

**Table 3:** Meta-regression analysis for primary and secondary outcomes (based on the primary analysis assumption, Corr=0.5).

Biomarker	Covariate	Trials	*β*-coefficient (95% CI)	*p*-value	R²	Residual I²	*p*-value for residual heterogeneity
BMI	Dosage_regimen	5	0.33 (-3.08; 3.74)	0.851	0.00%	0.00%	0.851
	Followup_duration_weeks	5	0.09 (-0.15; 0.33)	0.462	0.00%	0.00%	0.963
	Population_Grouped	5	0.33 (-3.08; 3.74)	0.851	0.00%	0.00%	0.851
HOMA_IR	Dosage_regimen	7	0.46 (-0.94; 1.85)	0.522	0.00%	0.00%	0.923
	Followup_duration_weeks	7	0.00 (-0.08; 0.08)	0.978	0.00%	0.00%	0.874
	Population_Grouped	7	1.12 (-1.25; 3.49)	0.355	0.00%	0.00%	0.966
HbA1c	Dosage_regimen	6	-0.56 (-1.09; -0.02)	0.040	92.71%	14.30%	0.332
	Followup_duration_weeks	6	0.03 (0.01; 0.05)	0.014	100.00%	0.00%	0.551
	Population_Grouped	6	-0.56 (-1.01; -0.10)	0.016	89.07%	19.45%	0.558
LDL	Dosage_regimen	5	0.39 (-0.52; 1.30)	0.395	0.00%	0.00%	0.596
	Followup_duration_weeks	5	0.01 (-0.01; 0.02)	0.318	0.00%	0.00%	0.656
	Population_Grouped	5	-0.17 (-0.65; 0.31)	0.489	0.00%	0.00%	0.546
TC	Dosage_regimen	5	0.58 (-0.42; 1.59)	0.255	0.00%	0.00%	0.853
	Followup_duration_weeks	5	-0.00 (-0.02; 0.01)	0.752	0.00%	0.00%	0.576
	Population_Grouped	5	0.30 (-0.31; 0.91)	0.338	0.00%	0.00%	0.762

### 3.5 Effects on HOMA-IR

A meta-analysis of seven trials showed that FMT intervention did not significantly improve HOMA-IR (MD: -0.64 [95% CI: -1.31, 0.03]; *p* = 0.062, I² = 4.1%, n = 211) (**Figure 3B**). Subgroup analyses (**Table 2**) revealed no significant differences based on control group type (*p* = 0.514), administration regimen (*p* = 0.221), follow-up duration (*p* = 0.465), or patient population (*p* = 0.064). Furthermore, meta-regression analysis revealed no significant association between changes in HOMA-IR and treatment regimen (*p* = 0.522), duration of follow-up (*p* = 0.978), or patient population (*p* = 0.355) (**Table 3**).

### 3.6 Effects on HbA1c

A total of 6 trials provided HbA1c data. The pooled analysis showed that FMT intervention did not reduce HbA1c levels, which were slightly but non-significantly higher compared to controls (MD: 0.06 [95% CI: -0.29; 0.40]; *p* = 0.742, I² = 59.0%, n = 156), with moderate heterogeneity between studies (**Figure 3C**). Subgroup analyses (**Table 2**) showed no statistically significant differences across subgroups, though a trend was observed in the follow-up duration subgroup (*p* = 0.056). In contrast, meta-regression analysis (**Table 3**) revealed significant linear associations between multiple factors and HbA1c change, including dosing regimen (*β* = -0.56; *p* = 0.040), follow-up duration (*β* = 0.03; *p* = 0.014), and patient population (*β* = -0.56; *p* = 0.016).

### 3.7 Effects on lipid profile

This analysis evaluated the overall effect of FMT on the lipid profile (**Figure 3D–F**). The pooled analysis showed that FMT intervention resulted in no significant changes in TC (MD: 0.03 [95% CI: -0.27; 0.32]; *p* = 0.848, n=134), LDL (MD: 0.18 [95% CI: -0.05; 0.42]; *p* = 0.120, n=134), HDL (MD: -0.05 [95% CI: -0.15; 0.06]; *p* = 0.380, n=113), or TG (MD: -0.21 [95% CI: -0.50; 0.08]; *p* = 0.163, n=113). Notably, no between-study heterogeneity was detected in all analyses (I² = 0.0%). Subgroup analyses (**Table 2**) and meta-regression analyses (**Table 3**) for all lipid parameters revealed no significant influence of any prespecified moderating variables (control group type, dosing regimen, follow-up duration, or patient population) on the results.

### 3.8 Sensitivity analysis

To assess the robustness of the results, we conducted a leave-one-out analysis and two sensitivity analyses based on different correlation coefficient assumptions. The leave-one-out analysis (**Supplementary Table S2**) revealed that for HOMA-IR, HbA1c, and all secondary lipid parameters, excluding any individual study sequentially, did not alter the statistical significance of the pooled results. For BMI, the pooled MD of the primary analysis was -0.65 (95% CI: -1.35, 0.05; *p* = 0.070). In the analysis excluding the study by Gómez-Pérez et al. (21), the pooled MD was -0.71 (95% CI: -1.42, 0.00; *p* = 0.050). In another analysis, we altered the baseline Corr value from 0.5 to 0.25 and 0.75 when estimating the standard deviation of effect size. Results showed that when assuming a correlation coefficient of 0.75, FMT intervention significantly reduced BMI (MD: -0.66 [95% CI: -1.16; -0.15]; *p* = 0.010). However, for HbA1c and all lipid markers, the statistical significance of the results remained unchanged across the three different correlation coefficient assumptions.

### 3.9 GRADE evidence assessment

According to the GRADE approach (**Supplementary Table S4**), the overall certainty of the current evidence for all primary and secondary outcome measures (BMI, HOMA-IR, HbA1c, and lipid profile) was rated as “Low.” Evidence grades were downgraded primarily due to “Risk of Bias” (based on methodological limitations assessed under RoB 2) and “Imprecision” (95% confidence intervals for pooled effect sizes encompassed non-significant values). A quantitative assessment of publication bias was not performed as fewer than 10 studies were included for each outcome measure.

## 4 Discussion

To our knowledge, this study represents one of the most recent and comprehensive systematic reviews and meta-analyses examining the efficacy of FMT in treating overweight or obese adults with metabolic disorders. In the primary analysis, our pooled results showed that allogeneic FMT intervention did not yield statistically significant improvements in primary outcome measures (BMI, HOMA-IR, HbA1c) or secondary lipid parameters compared to placebo or autologous FMT controls.

However, the evidence base for this overall negative conclusion remains weak. In our sensitivity analyses, outcomes for BMI exhibited high sensitivity to statistical estimation methods: adjusting the correlation coefficient (Corr) from 0.5 to 0.75 for estimating the standard deviation of change transformed FMT’s effect into statistical significance (*p* = 0.010) (**Supplementary Table S3**). The leave-one-out analysis (**Supplementary Table S2**) for BMI also demonstrated this; the primary pooled result was non-significant (*p* = 0.070), whereas the pooled result excluding the study by Gómez-Pérez et al. (21) was statistically significant (*p* = 0.050). This instability highlights the complexity of the current evidence base and suggests that the potential efficacy of FMT may be obscured by heterogeneity in study design and statistical methods.

Nevertheless, statistical significance does not equate to clinical relevance, and the magnitude of the observed effects warrants critical scrutiny. The pooled BMI reduction of 0.65 kg/m², while favoring intervention, remains below the 5–10% weight loss threshold typically required to improve cardiometabolic health (28). Similarly, the decrease in HOMA-IR (-0.64) offers limited benefit for severe insulin resistance, where normalization necessitates more substantial shifts. These findings imply that FMT’s current efficacy as a standalone monotherapy is modest compared to standard interventions. Thus, it may be more clinically viable as an adjunctive strategy rather than a primary treatment.

This finding of non-significant effects aligns with trends reported in existing systematic reviews in this field. Recent meta-analyses targeting metabolic syndrome have observed no significant improvement in HOMA-IR, lipid profiles, or BMI following FMT (29, 30). This suggests that under the current research paradigm, the overall efficacy of FMT in improving systemic insulin resistance remains to be established. However, the therapeutic efficacy of FMT in specific gastrointestinal disorders, such as ulcerative colitis (UC) and irritable bowel syndrome (IBS), is widely recognized (31, 32). This disparity in efficacy across indications may reflect differing intervention challenges: correcting systemic disorders like metabolic syndrome—involving multiple organs with complex pathophysiological networks and potentially more stable dysbiosis states—may present higher intervention barriers compared to localized intestinal inflammatory diseases such as UC or IBS.

1) First is the intensity and duration of the intervention. Our analysis indicates that the administration regimen (single vs. multiple doses) is significantly associated with changes in HbA1c (*p* = 0.040), with multiple-dose regimens linked to a greater trend toward HbA1c reduction (*β* = -0.56). This suggests that single-dose FMT interventions may struggle to sustain long-term effects in metabolic disease studies. This perspective is supported by the original research: Ghorban et al. (24) noted in their discussion that FMT’s positive effects diminished at 3 months, indicating that repeated transplants may be necessary to maintain the potential benefits. Similarly, follow-up duration was identified as a factor influencing HbA1c changes (*p* = 0.014). Craven et al. (15) also noted in exploratory analyses that FMT may require an extended time to potentially improve glucose and lipid metabolism disorders.

2) Second, differences in patient baseline characteristics. Meta-regression analysis revealed that patients’ baseline metabolic status (i.e., whether they were diabetic) was one factor influencing HbA1c changes (*p* = 0.016). A potential “floor effect” may exist in this study, where subjects had relatively well-controlled baseline metabolism, limiting the scope for FMT to produce significant improvements. For example, Yu et al. (16) and Gómez-Pérez et al. (21) both noted in their discussions that their subjects had only mild to moderate insulin resistance or well-controlled baseline metabolism. In contrast, Mocanu et al. (27) found that baseline hyperinsulinemia was an independent predictor of improved insulin resistance after FMT. Yu et al. (16) also observed, in their exploratory analysis, that FMT showed potential improvements in total cholesterol and HbA1c only in the subgroup with “low baseline microbial diversity.” Furthermore, Ng et al. (25) and Mocanu et al. (27) noted that FMT may require adjunctive measures to enhance efficacy, such as combined dietary interventions or high-fermentable fiber supplementation. These differences in study design collectively suggest that pooling data from studies with varying intervention frequencies, observation periods, participant baselines, and adjunctive measures may dilute or obscure positive effects present in specific subgroups.

3) Beyond factors quantified through meta-regression analysis, donor-related heterogeneity remains a key contributor to inconsistent FMT efficacy. Currently, included studies predominantly employ “healthy lean donors” as selection criteria, yet this functional definition exhibits considerable breadth. For instance, among the studies included in this meta-analysis, Witjes et al. (26) restricted donors to individuals following a plant-based diet, illustrating the lack of operational consistency in the “healthy” criterion. In recent years, the concept of “super-donors” has gained attention, suggesting that fecal microbiota from certain specific donors may yield superior clinical outcomes compared to others (33, 34). This variability is reflected in the included studies: Groenewegen et al. (20) observed that the downward trend in triglyceride levels was primarily driven by a single donor; Yu et al. (16) also reported significant “graft variability, “ where microbial communities from different donors exhibited differing colonization efficiency and persistence in recipients, potentially directly contributing to inconsistent metabolic responses. Therefore, combining data from donors with diverse functional backgrounds may result in potential positive effects being “averaged out” or diluted. Mocanu et al. (27) proactively assessed donor allocation across groups in their design to deliberately exclude interference from “super donor” phenomena, confirming that donor selection is a recognized key variable influencing FMT efficacy. Yu et al. (16) also recommended that future studies incorporate “donor-recipient pre-selection.”

From a mechanistic perspective, this variation in functional potential has been revealed in recent studies. Clinical response correlates with the successful engraftment of specific functional microbiota. For instance, one study linked weight loss in responders to the successful engraftment of Phascolarctobacterium genus and Acidaminococcaceae family members (both involved in short-chain fatty acid SCFA production) from donor sources (35). Moreover, the functional potential of donors extends beyond the production of beneficial substances like SCFAs. It also encompasses their ability to modulate bile acid profiles (e.g., converting primary bile acids into secondary bile acids, DCA and LCA) or clear harmful metabolites (e.g., ethanolamine), which are key signaling molecules regulating host glucose and lipid metabolism (36, 37). The donor’s own systemic metabolic phenotype has been found to potentially transfer to the recipient. A study by de Groot et al. (38) demonstrated that fecal transplantation from a metabolically impaired obese donor (METS-D) significantly worsened peripheral insulin sensitivity in recipients (*p* < 0.01). This finding was accompanied by deterioration in the recipient’s metabolome, notably elevated levels of secondary bile acids LCA and DCA. These findings suggest that an effective donor may require a functionally active microbiota-metabolite complex capable of correcting recipient-specific defects. Therefore, current donor screening criteria, which focus solely on excluding infectious pathogens, may be insufficient to ensure the efficacy of FMT in metabolic diseases (39).

4) Regarding administration routes, there is no clear consensus on an optimal approach. Among the trials included in this study, upper gastrointestinal routes (including oral capsules, nasojejunal tubes, and endoscopic infusion) were the primary methods, while the colonic route was less frequently employed. Different administration routes not only determine the initial colonization site and survival rate of the microbiota within the gut but may also introduce confounding factors. For example, Yu et al. (16) speculated that their oral capsule approach (without antibiotic pretreatment or bowel preparation) might be one reason for the lower graft efficiency and therapeutic differences compared to the endoscopic administration used in the Dutch study (12). da Ponte Neto et al. (22), who employed the colonoscopic route, noted that this approach “may not be optimal.” Craven et al. (15) further pointed out that even with duodenal administration, subsequent analysis of stool samples alone may not fully capture the actual microbial changes in the small intestine or proximal colon.

5) Key disagreements also exist regarding control group design. Autologous FMT and inert placebo are the two primary control forms. Autologous FMT is widely regarded as a stricter placebo because it more closely mimics the full experience of the transplant process. Although our subgroup analysis failed to detect statistically significant differences between control types due to the small number of studies within each group, this does not imply that such differences do not exist. The choice of placebo type may still influence trial outcomes (40).

## 5 Limitations

First, the certainty of evidence for this analysis was rated as “Low” according to the GRADE approach. The downgrading primarily stemmed from two aspects: (1) Risk of Bias: Among the 11 included RCTs, 9 (81.8%) were rated as having “some concerns.” These concerns were largely focused on the randomization process and deviations from intended interventions, potentially reflecting limitations in random sequence generation or suboptimal blinding efficacy, which may compromise the internal validity of individual trials. (2) Imprecision: The 95% confidence intervals for the pooled effect sizes of all primary outcomes spanned the null value, indicating that the current pooled data are insufficient to yield precise effect estimates.

Second, the robustness of this study’s primary conclusions is constrained by sensitivity to statistical assumptions. The negative result for BMI (*p* = 0.070) in the main analysis (based on Corr = 0.5) became statistically significant (*p* = 0.010) in the sensitivity analysis when the correlation coefficient was adjusted to 0.75 (**Supplementary Table S3**). The leave-one-out analysis (**Supplementary Table S2**) for BMI also demonstrated this; the primary pooled result was non-significant (*p* = 0.070), whereas the pooled result excluding the study by Gómez-Pérez et al. (21) was statistically significant (*p* = 0.050). This high sensitivity to statistical assumptions indicates that the negative conclusions of the primary analysis should not be interpreted as definitive evidence of FMT ineffectiveness, but rather suggest that current evidence relies heavily on statistical estimation methods.

Third, incomplete outcome reporting across the included RCTs restricted the data available for the pooled analysis. Consequently, effective sample sizes for the primary outcome (BMI) and secondary parameters (e.g., triglycerides) were reduced to only 5 and 4 trials, respectively. This likely left these specific analyses underpowered, potentially preventing the observed trends from reaching statistical significance. Therefore, future trials must prioritize the comprehensive reporting of these metabolic endpoints to allow for more robust conclusions.

Fourth, the variability of control interventions complicates the interpretation of our findings. The mix of autologous FMT, inert placebos, and active drugs (e.g., metformin) makes it challenging to assess the true efficacy of FMT. Notably, active comparators can improve outcomes in the control arm, thereby narrowing the performance gap between groups and potentially masking the intervention’s benefit. Future research should aim for consistent control protocols to ensure more reliable comparisons.

Finally, as fewer than 10 studies were included for each outcome measure, funnel plots or Egger’s test could not be used to quantitatively assess publication bias. Consequently, the potential impact of publication bias on the pooled effect size cannot be ruled out.

## 6 Conclusion

Based on current evidence of “low” certainty, this systematic review and meta-analysis indicate that FMT did not show overall statistically significant improvement in key metabolic indicators for overweight or obese patients with metabolic diseases in the primary analysis. However, this negative conclusion exhibits instability in sensitivity analyses (i.e., insufficient robustness) and is likely highly influenced by methodological heterogeneity (e.g., administration protocols) and donor functional variability. Therefore, the true effectiveness of FMT in this setting remains uncertain. Future trials should focus on precision medicine, standardizing donor selection, and optimizing administration protocols.

## Data Availability

The raw data supporting the conclusions of this article will be made available by the authors, without undue reservation.
