# Supplementary material for: Effects of fecal microbiota transplantation on glycemic and lipid profiles in overweight or obese patients with metabolic disorders: a systematic review and meta-analysis
Source: Front Endocrinol (Lausanne). 2025 Dec 15;16:1737543. doi: 10.3389/fendo.2025.1737543 (PMC12745229; doi:10.3389/fendo.2025.1737543)
Supplement: Supplementary file 1 [file Table1.docx]

Table S1. Detailed Search Strategies

| Database | Search strategy |
| --- | --- |
| Pubmed | ( "Fecal Microbiota Transplantation"[Mesh] OR "Fecal Microbiota Transplantation"[tiab] OR "Feces infus*"[tiab] OR "Fecal infus*"[tiab] OR "Microbiota infus*"[tiab] OR "Stool infus*"[tiab] OR "Microbiome infus*"[tiab] OR "Microflor infus*"[tiab] OR "Feces transplant*"[tiab] OR "Fecal transplant*"[tiab] OR "Microbiota transplant*"[tiab] OR "Stool transplant*"[tiab] OR "Microbiome transplant*"[tiab] OR "Microflor transplant*"[tiab] OR "Feces enema"[tiab] OR "Fecal enema"[tiab] OR "Microbiota enema"[tiab] OR "Stool enema"[tiab] OR "Microbiome enema"[tiab] OR "Microflor enema"[tiab] OR "Feces donor"[tiab] OR "Fecal donor"[tiab] OR "Microbiota donor"[tiab] OR "Stool donor"[tiab] OR "Microbiome donor"[tiab] OR "Microflor donor"[tiab] OR FMT[tiab] ) AND ( ( "Obesity"[Mesh] OR "Weight Loss"[MeSH Terms] OR "Obesity"[tiab] OR overweight[tiab] OR "Weight Loss"[tiab] OR "Losses, Weight"[tiab] OR "Loss, Weight"[tiab] OR "Weight Losses"[tiab] OR "Weight Reduction"[tiab] OR "Reductions, Weight"[tiab] OR "Reduction, Weight"[tiab] OR "Weight Reductions"[tiab] ) OR ( "Diabetes Mellitus, Type 2"[Mesh] OR "Type 2 Diabetes Mellitus"[tiab] OR "Diabetes Mellitus, Type 2"[tiab] OR T2DM[tiab] OR T2D[tiab] OR "Type 2 Diabetes"[tiab] OR "Diabetes, Type 2"[tiab] OR "Non-Insulin-Dependent Diabetes Mellitus"[tiab] OR NIDDM[tiab] OR "Adult-Onset Diabetes Mellitus"[tiab] OR "Maturity-Onset Diabetes Mellitus"[tiab] OR MODY[tiab] OR "Insulin Resistance"[Mesh] OR "insulin resistance"[tiab] OR "insulin sensitivity"[tiab] OR "impaired glucose tolerance"[tiab] OR IGT[tiab] OR "impaired fasting glucose"[tiab] OR IFG[tiab] OR prediabet*[tiab] OR "pre-diabet*"[tiab] OR HOMA-IR[tiab] OR "Matsuda Index"[tiab] OR "hyperinsulinemic euglycemic clamp"[tiab] ) OR ( "Metabolic Syndrome"[Mesh] OR "Metabolic Syndrome X"[tiab] OR "Insulin Resistance Syndrome X"[tiab] OR "Dysmetabolic Syndrome X"[tiab] OR "Metabolic Cardiovascular Risk Syndrome"[tiab] OR "Cardiometabolic Syndrome"[tiab] OR MetS[tiab] ) OR ( "Non-alcoholic Fatty Liver Disease"[Mesh] OR "Fatty Liver"[Mesh] OR "Metabolic Dysfunction-Associated Steatotic Liver Disease"[tiab] OR MASLD[tiab] OR "Non-alcoholic Fatty Liver Disease"[tiab] OR "Nonalcoholic Fatty Liver Disease"[tiab] OR NAFLD[tiab] OR "Non-alcoholic steatohepatitis"[tiab] OR "Nonalcoholic steatohepatitis"[tiab] OR NASH[tiab] OR "hepatic steatosis"[tiab] OR "liver fat"[tiab] ) OR ( "Hypertension"[Mesh] OR hypertension[tiab] OR "high blood pressure"[tiab] OR "elevated blood pressure"[tiab] OR hypertensive[tiab] ) ) AND ( "randomized controlled trial"[pt] OR "controlled clinical trial"[pt] OR randomized[tiab] OR placebo[tiab] OR randomly[tiab] OR assignment[tiab] OR trial[tiab] OR groups[tiab] OR intervention*[tiab] OR RCT[tiab] ) |
| Embase | ( 'fecal microbiota transplantation'/exp OR 'faecal microbiota transplantation':ab,ti OR 'fecal microbiota transplantation':ab,ti OR 'microbiome transfer':ab,ti OR 'microbiota transfer':ab,ti OR 'stool transplant*':ab,ti OR 'FMT':ab,ti OR 'Feces infus*':ab,ti OR 'Fecal infus*':ab,ti OR 'Microbiota infus*':ab,ti OR 'Stool infus*':ab,ti OR 'Microbiome infus*':ab,ti OR 'Microflor infus*':ab,ti OR 'Feces transplant*':ab,ti OR 'Fecal transplant*':ab,ti OR 'Microbiota transplant*':ab,ti OR 'Stool transplant*':ab,ti OR 'Microbiome transplant*':ab,ti OR 'Microflor transplant*':ab,ti OR 'Feces enema':ab,ti OR 'Fecal enema':ab,ti OR 'Microbiota enema':ab,ti OR 'Stool enema':ab,ti OR 'Microbiome enema':ab,ti OR 'Microflor enema':ab,ti OR 'Feces donor':ab,ti OR 'Fecal donor':ab,ti OR 'Microbiota donor':ab,ti OR 'Stool donor':ab,ti OR 'Microbiome donor':ab,ti OR 'Microflor donor':ab,ti ) AND ( ( 'obesity'/exp OR 'body weight loss'/exp OR 'obesity':ab,ti OR 'overweight':ab,ti OR 'weight loss':ab,ti OR 'weight reduction':ab,ti ) OR ( 'type 2 diabetes mellitus'/exp OR 'insulin resistance'/exp OR 'type 2 diabetes mellitus':ab,ti OR 'T2DM':ab,ti OR 'T2D':ab,ti OR 'non insulin dependent diabetes mellitus':ab,ti OR 'NIDDM':ab,ti OR 'adult onset diabetes mellitus':ab,ti OR 'maturity onset diabetes mellitus':ab,ti OR 'insulin resistance':ab,ti OR 'insulin sensitivity':ab,ti OR 'impaired glucose tolerance':ab,ti OR 'IGT':ab,ti OR 'impaired fasting glucose':ab,ti OR 'IFG':ab,ti OR 'prediabet*':ab,ti OR 'pre diabet*':ab,ti OR 'homa ir':ab,ti OR 'matsuda index':ab,ti ) OR ( 'metabolic syndrome'/exp OR 'metabolic syndrome':ab,ti OR 'metabolic syndrome x':ab,ti OR 'cardiometabolic syndrome':ab,ti OR 'MetS':ab,ti ) OR ( 'nonalcoholic fatty liver disease'/exp OR 'fatty liver'/exp OR 'metabolic dysfunction associated steatotic liver disease':ab,ti OR 'MASLD':ab,ti OR 'non alcoholic fatty liver disease':ab,ti OR 'nonalcoholic fatty liver disease':ab,ti OR 'NAFLD':ab,ti OR 'non alcoholic steatohepatitis':ab,ti OR 'nonalcoholic steatohepatitis':ab,ti OR 'NASH':ab,ti OR 'hepatic steatosis':ab,ti OR 'liver fat':ab,ti ) OR ( 'hypertension'/exp OR 'hypertension':ab,ti OR 'high blood pressure':ab,ti OR 'elevated blood pressure':ab,ti OR 'hypertensive':ab,ti ) ) AND ( 'randomized controlled trial'/exp OR 'randomization'/exp OR 'controlled clinical trial'/exp OR 'random*':ab,ti OR 'placebo':ab,ti OR 'trial':ab,ti OR 'assignment':ab,ti OR 'groups':ab,ti ) |
| Cochrane library | #1 - Intervention Concept (FMT) [mh "Fecal Microbiota Transplantation"] OR ("Fecal Microbiota Transplantation"):ti,ab,kw OR ("faecal microbiota transplantation"):ti,ab,kw OR ("microbiome transfer"):ti,ab,kw OR ("microbiota transfer"):ti,ab,kw OR (stool* NEAR/3 transplant*):ti,ab,kw OR (FMT):ti,ab,kw OR (feces NEAR/2 infus*):ti,ab,kw OR (fecal NEAR/2 infus*):ti,ab,kw OR (microbiota NEAR/2 infus*):ti,ab,kw OR (stool NEAR/2 infus*):ti,ab,kw OR (feces NEAR/2 enema):ti,ab,kw OR (fecal NEAR/2 enema):ti,ab,kw OR (microbiota NEAR/2 enema):ti,ab,kw OR (feces NEAR/2 donor):ti,ab,kw OR (fecal NEAR/2 donor):ti,ab,kw OR (microbiota NEAR/2 donor):ti,ab,kw OR (stool NEAR/2 donor):ti,ab,kw  #2 - Population Concept (Obesity & Weight) [mh Obesity] OR [mh "Weight Loss"] OR (obesity):ti,ab,kw OR (overweight):ti,ab,kw OR ("weight loss"):ti,ab,kw OR ("weight reduction"):ti,ab,kw  #3 - Population Concept (Diabetes & Insulin Resistance) [mh "Diabetes Mellitus, Type 2"] OR [mh "Insulin Resistance"] OR ("Type 2 Diabetes"):ti,ab,kw OR (T2DM):ti,ab,kw OR (NIDDM):ti,ab,kw OR ("insulin resistance"):ti,ab,kw OR ("insulin sensitivity"):ti,ab,kw OR (prediabet*):ti,ab,kw OR ("impaired glucose tolerance"):ti,ab,kw OR (HOMA-IR):ti,ab,kw  #4 - Population Concept (Metabolic Syndrome) [mh "Metabolic Syndrome"] OR ("Metabolic Syndrome"):ti,ab,kw OR (MetS):ti,ab,kw OR ("cardiometabolic"):ti,ab,kw  #5 - Population Concept (Fatty Liver) [mh "Non-alcoholic Fatty Liver Disease"] OR [mh "Fatty Liver"] OR ("Metabolic Dysfunction-Associated Steatotic Liver Disease"):ti,ab,kw OR (MASLD):ti,ab,kw OR ("Non-alcoholic Fatty Liver Disease"):ti,ab,kw OR (NAFLD):ti,ab,kw OR ("Non-alcoholic steatohepatitis"):ti,ab,kw OR (NASH):ti,ab,kw OR ("hepatic steatosis"):ti,ab,kw OR ("liver fat"):ti,ab,kw  #6 - Population Concept (Hypertension) [mh Hypertension] OR (hypertension):ti,ab,kw OR ("high blood pressure"):ti,ab,kw OR (hypertensive):ti,ab,kw  #7 - Combined Population Concepts #2 OR #3 OR #4 OR #5 OR #6  #8 - Study Design Concept (RCT) [mh "Randomized Controlled Trials"] OR (randomized):ti,ab,kw OR (placebo):ti,ab,kw OR (randomly):ti,ab,kw OR (trial):ti,ab,kw OR (groups):ti,ab,kw  #9 - Final Combination #1 AND #7 AND #8 |
| Web of Science | TS=("Fecal Microbiota Transplantation" OR "Faecal Microbiota Transplantation" OR "Microbiome Transfer" OR "Microbiota Transfer" OR "Stool Transplant*" OR FMT OR "Feces infus*" OR "Fecal infus*" OR "Microbiota infus*" OR "Stool infus*" OR "Feces enema" OR "Fecal enema" OR "Microbiota enema" OR "Feces donor" OR "Fecal donor" OR "Microbiota donor" OR "Stool donor") AND TS=(Obesity OR Overweight OR "Weight Loss" OR "Weight Reduction" OR "Body Mass Index" OR BMI OR "Metabolic Syndrome" OR MetS OR "Type 2 Diabetes" OR T2DM OR "Insulin Resistance" OR "Insulin Sensitivity" OR Prediabet* OR "Impaired Glucose Tolerance" OR "Non-alcoholic Fatty Liver Disease" OR "Nonalcoholic Fatty Liver Disease" OR NAFLD OR "Metabolic Dysfunction-Associated Steatotic Liver Disease" OR MASLD OR "Hepatic Steatosis" OR Hypertension OR "High Blood Pressure") AND (TI=(randomized OR randomised OR placebo OR "controlled trial") OR TS=(randomly OR trial OR groups OR assignment OR intervention*)) |

Table S2. Consolidated Leave-One-Out Sensitivity Analysis (corr = 0.5)

| Outcome | Omitted Study / Overall | MD (95% CI) | I² (%) |
| --- | --- | --- | --- |
| BMI | Craven_2020 | -0.65 [-1.35, 0.06] | 0.0 |
|  | Ghorbani_2022 | -0.65 [-1.36, 0.05] | 0.0 |
|  | Gomez-Perez_2024 | -0.71 [-1.42, 0.00] | 0.0 |
|  | Vrieze_2012 | -0.66 [-1.37, 0.05] | 0.0 |
|  | Wu_2023 | 0.28 [-2.18, 2.74] | 0.0 |
|  | Overall | -0.65 [-1.35, 0.05] | 0.0 |
| HOMA_IR | Craven_2020 | -0.64 [-1.33, 0.06] | 20.1 |
|  | Ghorbani_2022 | -0.85 [-1.81, 0.12] | 15.9 |
|  | Gomez-Perez_2024 | -0.61 [-1.29, 0.07] | 16.1 |
|  | Wu_2023 | -0.38 [-1.08, 0.33] | 0.0 |
|  | Mocanu_2021 | -0.85 [-1.91, 0.22] | 18.3 |
|  | Ng_2022 | -0.65 [-1.34, 0.03] | 19.3 |
|  | Yu_2020 | -0.87 [-1.78, 0.05] | 10.8 |
|  | Overall | -0.64 [-1.31, 0.03] | 4.1 |
| HbA1c | Ghorbani_2022 | 0.08 [-0.44, 0.61] | 67.0 |
|  | Gomez-Perez_2024 | -0.05 [-0.21, 0.12] | 44.0 |
|  | Wu_2023 | 0.13 [-0.21, 0.47] | 55.8 |
|  | Ng_2022 | -0.00 [-0.37, 0.36] | 61.8 |
|  | Yu_2020 | 0.15 [-0.29, 0.59] | 60.0 |
|  | daPonteNeto_2023 | 0.05 [-0.34, 0.44] | 66.9 |
|  | Overall | 0.06 [-0.29, 0.40] | 59.0 |
| HDL_cholesterol | Ghorbani_2022 | -0.03 [-0.16, 0.10] | 0.0 |
|  | Vrieze_2012 | -0.07 [-0.19, 0.04] | 0.0 |
|  | Wu_2023 | -0.04 [-0.16, 0.08] | 0.0 |
|  | Ng_2022 | -0.04 [-0.17, 0.09] | 0.0 |
|  | Overall | -0.05 [-0.15, 0.06] | 0.0 |
| LDL_cholesterol | Ghorbani_2022 | 0.24 [-0.03, 0.51] | 0.0 |
|  | Vrieze_2012 | 0.21 [-0.03, 0.45] | 0.0 |
|  | Wu_2023 | 0.19 [-0.05, 0.44] | 0.0 |
|  | Ng_2022 | 0.08 [-0.24, 0.40] | 0.0 |
|  | Wijtjes_2020 | 0.16 [-0.08, 0.40] | 0.0 |
|  | Overall | 0.18 [-0.05, 0.42] | 0.0 |
| Total_cholesterol | Ghorbani_2022 | -0.01 [-0.34, 0.33] | 0.0 |
|  | Vrieze_2012 | 0.02 [-0.29, 0.33] | 0.0 |
|  | Wu_2023 | 0.07 [-0.24, 0.39] | 0.0 |
|  | Ng_2022 | 0.08 [-0.33, 0.49] | 0.0 |
|  | Wijtjes_2020 | -0.00 [-0.31, 0.30] | 0.0 |
|  | Overall | 0.03 [-0.27, 0.32] | 0.0 |
| Triglycerides | Ghorbani_2022 | -0.08 [-0.62, 0.45] | 0.0 |
|  | Vrieze_2012 | -0.22 [-0.52, 0.09] | 0.0 |
|  | Wu_2023 | -0.19 [-0.48, 0.11] | 0.0 |
|  | Ng_2022 | -0.27 [-0.59, 0.06] | 0.0 |
|  | Overall | -0.21 [-0.50, 0.08] | 0.0 |

Table S3. Sensitivity Analysis Based on Different Correlation (Corr) Assumptions)

| Outcome | Correlation Assumption | Trials | Participants | MD (95% CI) | *p*-value | I² |
| --- | --- | --- | --- | --- | --- | --- |
| BMI | corr = 0.50 | 5 | 107 | -0.65 (-1.35, 0.05) | 0.070 | 0.0% |
|  | corr = 0.75 | 5 | 107 | -0.66 (-1.16, -0.15) | 0.010 | 0.0% |
|  | corr = 0.25 | 5 | 107 | -0.65 (-1.50, 0.21) | 0.139 | 0.0% |
| HOMA_IR | corr = 0.50 | 7 | 211 | -0.64 (-1.31, 0.03) | 0.062 | 4.1% |
|  | corr = 0.75 | 7 | 211 | -0.77 (-1.58, 0.03) | 0.059 | 45.3% |
|  | corr = 0.25 | 7 | 211 | -0.65 (-1.46, 0.16) | 0.117 | 0.0% |
| HbA1c | corr = 0.50 | 6 | 156 | 0.06 (-0.29, 0.40) | 0.742 | 59.0% |
|  | corr = 0.75 | 6 | 156 | 0.06 (-0.36, 0.48) | 0.791 | 78.6% |
|  | corr = 0.25 | 6 | 156 | -0.00 (-0.01, 0.01) | 0.994 | 39.5% |
| HDL_cholesterol | corr = 0.50 | 4 | 113 | -0.05 (-0.15, 0.06) | 0.380 | 0.0% |
|  | corr = 0.75 | 4 | 113 | -0.05 (-0.12, 0.03) | 0.224 | 0.0% |
|  | corr = 0.25 | 4 | 113 | -0.05 (-0.18, 0.08) | 0.471 | 0.0% |
| LDL_cholesterol | corr = 0.50 | 5 | 134 | 0.18 (-0.05, 0.42) | 0.120 | 0.0% |
|  | corr = 0.75 | 5 | 134 | 0.18 (-0.01, 0.36) | 0.057 | 0.0% |
|  | corr = 0.25 | 5 | 134 | 0.18 (-0.10, 0.46) | 0.201 | 0.0% |
| Total_cholesterol | corr = 0.50 | 5 | 134 | 0.03 (-0.27, 0.32) | 0.848 | 0.0% |
|  | corr = 0.75 | 5 | 134 | 0.03 (-0.19, 0.24) | 0.815 | 0.0% |
|  | corr = 0.25 | 5 | 134 | 0.03 (-0.32, 0.38) | 0.869 | 0.0% |
| Triglycerides | corr = 0.50 | 4 | 113 | -0.21 (-0.50, 0.08) | 0.163 | 0.0% |
|  | corr = 0.75 | 4 | 113 | -0.20 (-0.42, 0.03) | 0.083 | 0.0% |
|  | corr = 0.25 | 4 | 113 | -0.21 (-0.56, 0.13) | 0.226 | 0.0% |

Table S4: GRADE Summary of Findings - Effect of FMT on Metabolic Outcomes

| Outcome | Studies | Participants | Effect Measure | Effect Size (95% CI) | Risk of Bias | Inconsistency | Imprecision | Publication Bias | Certainty of Evidence | Reasons for Rating Down |
| --- | --- | --- | --- | --- | --- | --- | --- | --- | --- | --- |
| BMI | 5 | 107 | MD | -0.65 (-1.35, 0.05) | Serious (-1) | Not serious | Serious (-1) | Not assessed | Low | Risk of Bias: All 5 studies had 'Some concerns' RoB. Inconsistency: I² = 0.0%, indicating no heterogeneity. Imprecision: 95% CI crosses the line of no effect (p=0.070). Publication Bias: Not assessed due to <10 studies. |
| HOMA-IR | 7 | 211 | MD | -0.64 (-1.31, 0.03) | Serious (-1) | Not serious | Serious (-1) | Not assessed | Low | Risk of Bias: Majority of studies (at least 5/7) had 'Some concerns' RoB. Inconsistency: I² = 4.1%, indicating low heterogeneity. Imprecision: 95% CI crosses the line of no effect (p=0.062). Publication Bias: Not assessed due to <10 studies. |
| HbA1c | 6 | 156 | MD | 0.06 (-0.29, 0.40) | Serious (-1) | Not serious | Serious (-1) | Not assessed | Low | Risk of Bias: Majority of studies (at least 5/6) had 'Some concerns' RoB. Inconsistency: I² = 59.0%, indicating moderate but not substantial heterogeneity. Imprecision: 95% CI widely crosses the line of no effect (p=0.742). Publication Bias: Not assessed due to <10 studies. |
| HDL | 4 | 113 | MD | -0.05 (-0.15, 0.06) | Serious (-1) | Not serious | Serious (-1) | Not assessed | Low | Risk of Bias: Majority of overall evidence (9/11 studies) had 'Some concerns' RoB. Inconsistency: I² = 0.0%, indicating no heterogeneity. Imprecision: 95% CI crosses the line of no effect (p=0.380). Publication Bias: Not assessed due to <10 studies. |
| LDL | 5 | 134 | MD | 0.18 (-0.05, 0.42) | Serious (-1) | Not serious | Serious (-1) | Not assessed | Low | Risk of Bias: Majority of overall evidence (9/11 studies) had 'Some concerns' RoB. Inconsistency: I² = 0.0%, indicating no heterogeneity. Imprecision: 95% CI crosses the line of no effect (p=0.120). Publication Bias: Not assessed due to <10 studies. |
| TC | 5 | 134 | MD | 0.03 (-0.27, 0.32) | Serious (-1) | Not serious | Serious (-1) | Not assessed | Low | Risk of Bias: Majority of overall evidence (9/11 studies) had 'Some concerns' RoB. Inconsistency: I² = 0.0%, indicating no heterogeneity. Imprecision: 95% CI widely crosses the line of no effect (p=0.848). Publication Bias: Not assessed due to <10 studies. |
| TG | 4 | 113 | MD | -0.21 (-0.50, 0.08) | Serious (-1) | Not serious | Serious (-1) | Not assessed | Low | Risk of Bias: Majority of overall evidence (9/11 studies) had 'Some concerns' RoB. Inconsistency: I² = 0.0%, indicating no heterogeneity. Imprecision: 95% CI crosses the line of no effect (p=0.163). Publication Bias: Not assessed due to <10 studies. |
